# How Structural and Physicochemical Determinants Shape Sequence Constraints in a Functional Enzyme

**DOI:** 10.1371/journal.pone.0118684

**Published:** 2015-02-23

**Authors:** Luciano A. Abriata, Timothy Palzkill, Matteo Dal Peraro

**Affiliations:** 1 Laboratory for Biomolecular Modeling, School of Life Sciences, and Swiss Institute of Bioinformatics, École Polytechnique Fédérale de Lausanne, Lausanne, Switzerland; 2 Department of Pharmacology, Baylor College of Medicine, BCM-385, Houston, Texas, United States of America; University of South Florida College of Medicine, UNITED STATES

## Abstract

The need for interfacing structural biology and biophysics to molecular evolution is being increasingly recognized. One part of the big problem is to understand how physics and chemistry shape the sequence space available to functional proteins, while satisfying the needs of biology. Here we present a quantitative, structure-based analysis of a high-resolution map describing the tolerance to all substitutions in all positions of a functional enzyme, namely a TEM lactamase previously studied through deep sequencing of mutants growing in competition experiments with selection against ampicillin. Substitutions are rarely observed within 7 Å of the active site, a stringency that is relaxed slowly and extends up to 15–20 Å, with buried residues being especially sensitive. Substitution patterns in over one third of the residues can be quantitatively modeled by monotonic dependencies on amino acid descriptors and predictions of changes in folding stability. Amino acid volume and steric hindrance shape constraints on the protein core; hydrophobicity and solubility shape constraints on hydrophobic clusters underneath the surface, and on salt bridges and polar networks at the protein surface together with charge and hydrogen bonding capacity. Amino acid solubility, flexibility and conformational descriptors also provide additional constraints at many locations. These findings provide fundamental insights into the chemistry underlying protein evolution and design, by quantitating links between sequence and different protein traits, illuminating subtle and unexpected sequence-trait relationships and pinpointing what traits are sacrificed upon gain-of-function mutation.

## Introduction

The amino acid sequence of a protein defines its structure and function, but also a number of other entangled traits such as stability, water solubility/membrane integration, potential for interactions with other molecules, internal dynamics, hydration (referring to water molecules wetting the protein in its first solvation sphere and cavities), etc. These traits arise as a complex combination of the basic physicochemical properties of the constituent amino acids, in a way related to the ordered context of the protein’s sequence. Given that all amino acids are chemically different from each other, all substitutions are expected to affect at least some of the traits associated to a particular protein sequence. If the effects produced by a substitution are sufficiently detrimental, the reduced function of the resulting protein will lead to decreased fitness and the mutation will likely not propagate in a population; on the contrary, if the effects are positive the mutation may increase in frequency and possibly be fixed (*i*.*e*. become permanent in the population). Thus, the physicochemical properties of the amino acids, and how they interact across a sequence, define not only the basic traits of a protein but also the potential of these traits to change and of the protein to evolve [1–3]. Whereas models of protein evolution account at most for protein structure at a coarse level [4–9], a fine, detailed understanding of how protein sequences evolve requires a full understanding of the effects of substitutions on all traits, to be incorporated into the evolutionary models [2,10–12]. This calls for a link between studies of genetic variation and structural biology [11].

From the side of structural biology, the problem fits into the goal of understanding the fundamental physical and chemical constraints that shape the sequence space available to functional proteins [11,13]. The most common approach towards capturing the relationships between protein sequence, structure, function and other traits has been to probe the effects of specific amino acid substitutions. Such studies, carried out on hundreds of proteins, have indeed provided most of our knowledge about protein physics and chemistry. However, they are inherently low-throughput and heterogeneous in terms of the recorded observables and the employed methods and materials. The new era of high-throughput deep sequencing technologies has allowed for much more complex experiments in which large libraries of mutants can be screened and then sequenced at once [14–17]. These technologies have been applied to full scans of a few proteins in studies about tolerance to substitution and distribution of fitness effects [18–22] and to monitor directed evolution experiments [23,24]. The produced data could provide at least part of the information required to connect genetic variability to structural biology [25–27] and to guide the development of better protein evolutionary models [28].

Based on deep sequencing technologies, a pioneering work by Deng et al. reported what is probably the highest-resolution map of an enzyme’s tolerance to amino acid substitutions across its entire sequence [18]. That work quantified the likelihood of substituting each residue of TEM-1 β-lactamase by each of the other 19 amino acids while retaining enzymatic activity in the cell against ampicillin. As discussed in the original paper, the experiment revealed that TEM-1 is quite tolerant of amino acid substitutions, except in the region that surrounds the active site and other positions scattered throughout the protein. The low tolerance to substitutions around the active site was interpreted by Deng et al. on the basis of catalytic and/or substrate-binding roles for these residues, whereas tolerance farther away from the active site was attributed to general roles in conferring stability and solubility. As we show here, the high resolution of this dataset allows the retrieval of richer and finer quantitative information about the physical and chemical basis for why different amino acids are more or less favored at each position of the protein, at least in the context of ampicillin hydrolysis by TEM-1. We have analyzed the substitution patterns determined for each position of TEM-1 on the basis of its three-dimensional structure and considering which physicochemical properties of the amino acids could dictate the observed distributions. Expanding on the observation that the active site can barely tolerate substitutions, we found that this stringency is still strong within 5–10 Å of the active site, with upper limits that relax only slowly as the distance increases and extending up to 15–20 Å away from it. We also found that the substitution patterns observed for over one third of the 263 residues of mature TEM-1 is quantitatively explained by monotonic dependencies on simple amino acid descriptors and through predictions of stability changes. Most of the explained patterns reflect effects on stability of the protein core, stability of hydrophobic clusters beneath the surface, stability of polar networks and salt bridges on the protein surface, amino acid solubility and backbone flexibility. Other descriptors point at very specific features related to backbone conformations or even substrate binding and catalysis themselves. Notably, most of the wild type residues are already (nearly) optimal, corresponding to (nearly) optimal values of the relevant descriptors. Therefore, mutations will tend to be detrimental regarding the relevant descriptor at each site, posing important consequences on the physicochemical aspects of protein evolution and protein design.

## Results and Discussion

The information produced by Deng et al. is available in the form of ΔΔG_i,j_^stat^, which measures the frequency of each amino acid *j* at each position *i* of TEM-1’s sequence relative to the frequency of the wild type residue, and *k*_i_* which quantifies the effective number of amino acids tolerated at each position *i*. The k* parameter runs from 1 for a position fully restricted to one amino acid to 20 for a position in which any amino acid is equally likely. In turn, ΔΔG^stat^ is by definition zero for the wild type amino acid at a given position, negative for those substitutions that appear at a higher frequency than the wild type amino acid and positive for those at lower frequency than wild type.

### Residues within 10 Å of the active site or with less than 20% of their surface exposed to the solvent experience strong constraints on amino acid identity

**Fig 1:**
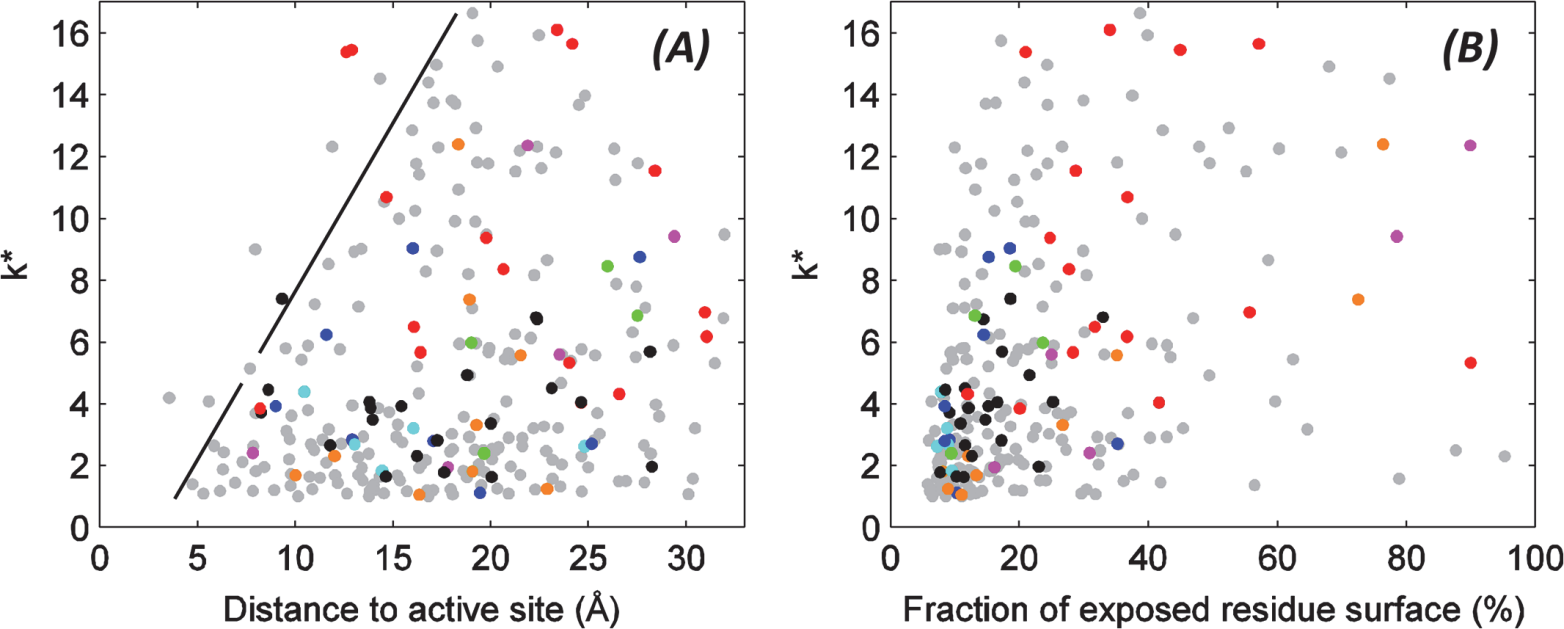
Effective number of substitutions versus distance to the active site and solvent exposure. Effective number of amino acids that appear at each position (k*) plotted versus the distance from its Cα atom to the active site (A) and against the fraction of amino acid surface exposed to the solvent (B). The position of the active site was defined as the average position of the Ser70, Lys73 and Glu166 Cα atoms; surface exposure was computed with the POPS webserver [48,49] based on the X-ray structure deposited under PDB entry 1XPB [50]. Dot colors correspond to correlations against the most often matched properties: blue for *volume*, cyan for *volume/(P(helix)+P(sheet))*, green for *steric hindrance*, dark green for *steric hindrance / P(sheet)*, red for *hydrophobicity*, magenta for *log(solubility) x hydrophobicity*, orange for *flexibility x hydrophobicity*. Black dots represent correlations with FoldX predictions. Gray dots correspond to the rest of the residues (with other or no detected correlations). The line drawn in panel A arbitrarily indicates how the maximum possible k* increases with distance to the active site.

We first tested quantitatively the observation by Deng et al. that TEM-1 is generally tolerant of substitutions except in the surroundings of the active site and in distant positions that presumably confer solubility and stability. A plot of k* against distance to the active site for all residues (Fig. 1A, where the distance of a given residue to the active site is defined as the distance from its Cα to the average coordinates of Ser70’s, Lys73’s and Glu166’s Cα atoms) reveals that within a radius of 7 Å, each wild type residue tolerates substitutions by other 2–4 amino acids at most (k* < 5). Even within 10 Å, all but two positions have k* < 6, the two outliers being still below 10. The 7 Å sphere includes 9 residues into the “very sensitive” region surrounding the active site, whereas 22 additional residues lie between 7 and 10 Å. Beyond the 10 Å sphere the maximum observed k* values rise slowly, crossing the limit of 15 for distances longer than 12.5 Å (24 additional residues). Still, within ∼20 Å of the active site (additional 110 residues) there is a higher density of points at low k* values, and only at longer distances is the distribution of k* values smoother. In summary, tight constraints shape the protein’s sequence within 10 Å of its active site, and they are only slowly relaxed beyond that limit, thus extending to large numbers of residues. This “relaxation” region is approximated in Fig. 1A with a black line that arbitrarily indicates an upper limit of tolerance to substitutions. The slope of this line is ∼1.1 Å^-1^, suggesting that roughly one additional amino acid can substitute for the wild type amino acid for every Å progressing away from the active site, at least for this dataset specific to TEM-1 hydrolyzing ampicillin. The long-reaching effect of the active site agrees with the finding, in several systems including lactamases, that interactions from the second shell and beyond very often fine-tune protein function [29–33].

The plot in Fig. 1B, which depicts k* versus the fraction of exposed surface area for each residue, shows as a main trend a higher density of points in the region with very low exposed surface and very low k*: 62.8% of the residues have less than 20% of their surface areas exposed, 75.9% of which have k* < 5. This highlights strict amino acid constraints in most of the protein core, as pointed out by Deng et al. for ampicillin hydrolysis by TEM-1 but consistent with several other studies suggesting this is a rather general effect [3,34,35]. For residues with more than 17% of their surface areas exposed, the smooth distribution of k* values indicates variable tolerance to substitutions.

Notably, the maximum k* of 16–17 implies that for any site in the protein, there are always at least 3 or 4 amino acids that are very strongly disallowed. As discussed by Deng et al., tryptophan and proline are the two least tolerated amino acids, bearing the largest ΔΔG^stat^ averages across the sequence and being favored only when they are the native amino acid. This metric can be expanded by computing for each amino acid the average ΔΔG^stat^ across sites where they are nonnative, which yields Trp, Pro, Gly, Phe, Cys, Glu and Arg as the least tolerated amino acids with values of 3.39, 3.23, 3.04, 3.02, 2.96, 2.92 and 2.85. (The average over sites where each amino acid is nonnative differs by > 0.2 from the average over the full sequence for Ala, Glu, Gly and Leu, and by 0.1–0.2 for Asp, Ile, Lys, Pro, Arg, Ser, Thr and Val, altering the relative order of amino acids in the list of least tolerated ones. For example, Gly ranks 5th across the full sequence but 3rd across nonnative sites, and Tyr ranks 7th across the full sequence but 9th across nonnative sites.). This trend argues against substitutions by these residues when designing mutations (for comparison, the other amino acids span ΔΔG^stat^ from 2.29 for Thr to 2.74 for Lys across sites where they are nonnative). Quoting Deng et al., the negative impact of substitutions by Trp may stem from steric clashes of its large side chain, whereas the negative impact of Pro could arise from effects on backbone conformation especially disruption of helical structures. Likewise, Phe can be hard to accommodate due to its hydrophobicity (notably, the more polar Tyr is well tolerated, so a size factor is in principle less important for these two amino acids); Gly can introduce flexibility in the backbone and/or produce void spaces that destabilize the structure; whereas cysteine can foster covalent dimerization or be prone to oxidation introducing large negative charges and possibly formation of reactive free radicals.

### Finding out the amino acid descriptors that best explain the observed ΔΔG^stat^ distributions

We then attempted to rationalize the observed ΔΔG^stat^ distributions on the basis of the physicochemical properties of the amino acids. A set of 28 descriptors was assembled that describes different properties of the amino acids with low correlation to each other (S1 Table), based on previous works that developed minimal complete sets of amino acid properties [36,37] and combinations of them. The descriptors were evaluated for those that could explain through linear correlations the distribution of ΔΔG^stat^ values observed at each position of TEM-1 for ampicillin hydrolysis. Dependencies were selected when they produced correlations better than r = 0.67 (r^2^ = 0.45) and RMSD < 1 between experimental and linearly back-predicted ΔΔG^stat^ values, as a proxy for monotonicity. The exact procedure and list of descriptors are provided in Methods.

**Fig 2:**
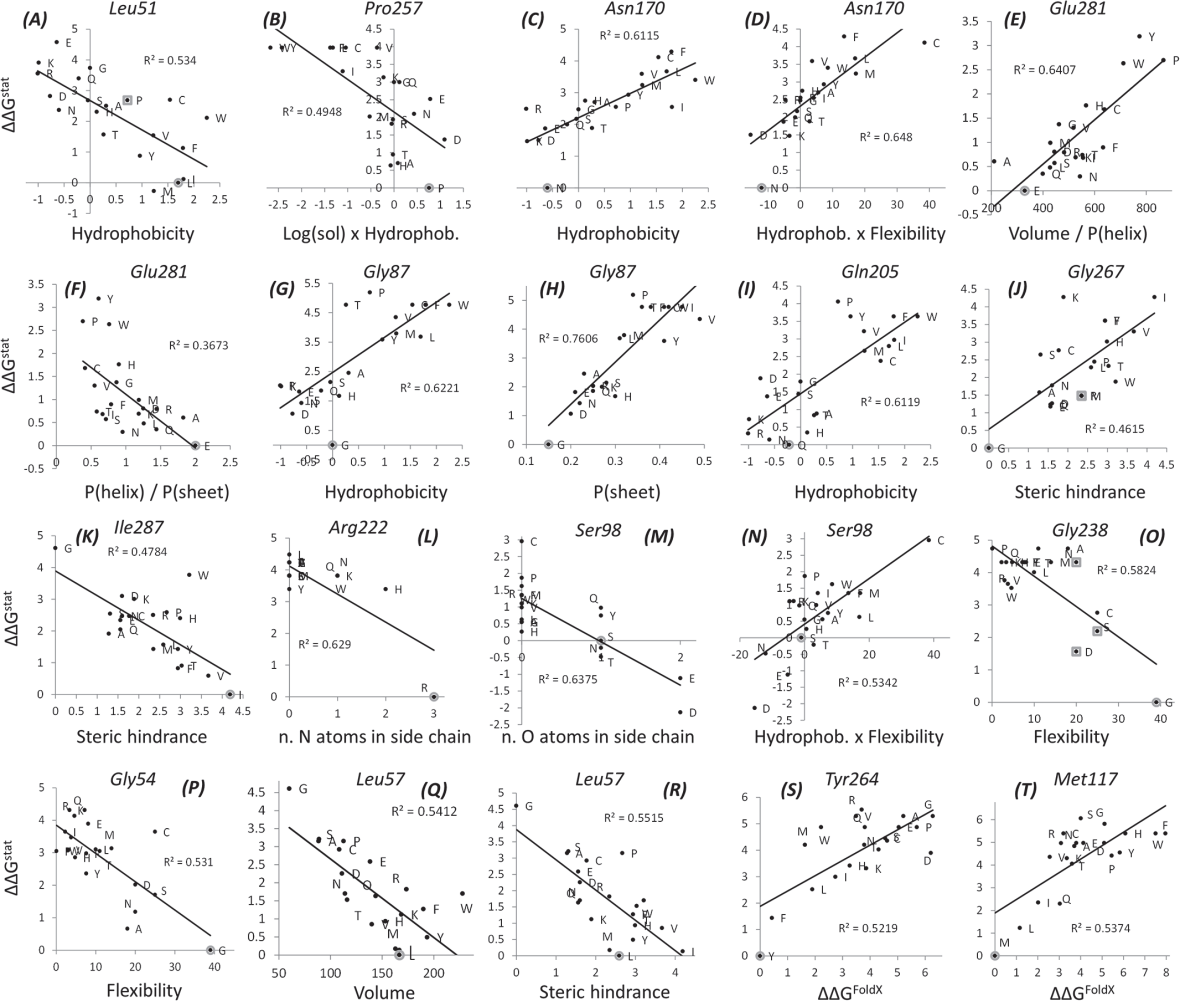
Examples of correlations detected between ΔΔG^stat^ values and amino acid descriptors (a-r) or ΔΔG^FoldX^ (s-t). The gray circles point at the wild type amino acid, gray squares point at substitutions that have been observed in natural TEM variants. Lines correspond to best linear fits.

A total of 105 correlations were obtained between ΔΔG^stat^ values and amino acid descriptors, 73 of which are best correlations (*i*.*e*. they map to 73 different residues) and 32 are second-best correlations (*i*.*e*. they satisfy the r and RMSD criteria but slightly less well than another, best correlation). Of the 28 descriptors, 20 could fit at least one site. *Hydrophobicity*, *Volume* and *log(solubility) x Hydrophobicity* were those most often selected by the procedure, accounting for almost one third of the total (S1 Fig.). Nine additional descriptors were selected more than five times: number of O atoms in the side chain, number of N atoms in the side chain, *Volume / log(solubility)*, *P(helix) + P(sheet)* (standing for alpha helical and beta sheet propensities, respectively), *Volume / P(helix)*, *Flexibility* (sensitive to backbone flexibility), *Hydrophobicity x Flexibility*, *Volume / (P(helix) + P(sheet))* and *Steric hindrance*. Examples of correlations between ΔΔG^stat^ and the corresponding descriptors are shown in Fig. 2 (A-R) and discussed in the next subsection. All the correlations are mapped to the sequence in S2 Fig. and can be explored interactively in the spreadsheet provided in the supporting material (S2 Table).

Given the important role of stability constraints in shaping proteins, we further scanned the ΔΔG^stat^ distributions against the changes in folding stability predicted by the program FoldX [38] for each of the 19 mutants at each position of TEM-1 (ΔΔG^FoldX^ values). Like other programs of its kind, FoldX performs poorly at quantitatively reproducing experimental changes in stability but is considered to correctly reflect trends [34,35,39,40], which could potentially fit our goal by helping to explain the ΔΔG^stat^ distributions of residues under complex constraints. The search for correlations between ΔΔG^stat^ and ΔΔG^FoldX^ values results in 25 residue positions for which r > 0.67 (S3 Table). All these residues form portions of hydrophobic clusters inside the protein core, indicating that FoldX is especially good at identifying them (*i*.*e*. black points in Fig. 1B). Only five of them overlap with those to which descriptors were mapped above, hence we can add 20 new explained distributions to our list to reach a total of 93 residue sites for which descriptors accurately model experimental results. Examples of selected dependencies between ΔΔG^stat^ and ΔΔG^FoldX^ are given in Fig. 2S and 2T and discussed below; all of them are available as an interactive spreadsheet in S4 Table.

### A third of the amino acid substitutions tolerated by TEM-1 can be quantitatively modeled by the amino acid descriptors

The protocol employed to search for relationships between ΔΔG^stat^ distributions and amino acid descriptors assumes dependencies on only one property, or two at most in the case of composite variables, and that the involved relationships are close to linear. Despite its simplicity, though, it allowed us to quantitatively explain, together with FoldX predictions, over 35% of the observed substitution patterns. This means that the identities of the amino acids in approximately one third of the TEM-1 sequence are shaped mainly by one or two fundamental properties in a monotonic fashion and by well-captured effects on stability, whereas other variables have much less weight in defining the most likely residues at those positions.

**Fig 3:**
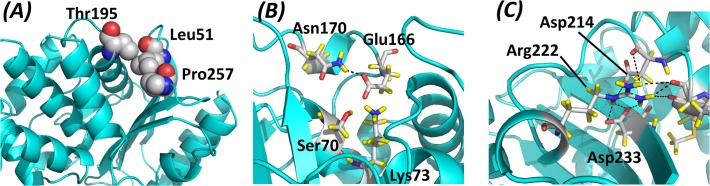
Structural representation of the (a) Leu51 (b) Asn170 (c) Arg222 regions in the TEM-1 β-lactamase structure. Pictures rendered from PDB ID 1XPB [50] using the program PyMOL [51]. Atom colors are red for oxygen, blue for nitrogen, gray for carbon and yellow for hydrogen.

Besides the general conclusions outlined in the next subsection, the correlation plots can be interpreted in detail. For example, Leu51, whose side chain forms part of a small hydrophobic cluster with the methyl group of Thr195 and the Pro257 side chain directly beneath the protein surface (Fig. 3A), is sensitive to hydrophobicity favoring more hydrophobic residues (Fig. 2A). Position 257 (Fig. 2B) responds to *log(solubility) x Hydrophobicity*, which means that amino acids simultaneously hydrophobic (to pack with Leu51) and soluble (possibly because polar side chains at this position could snorkel out of the protein) are favored. No property was selected for Thr195, due to the small dispersion of ΔΔG^stat^ values at that position (0.78 against average and median deviations of 1.14 and 1.15 across the full sequence) and its high tolerance to substitution (k* = 15.9). These are examples of constraints that act to stabilize superficial hydrophobic clusters, as opposed to those clusters truly at the protein core which are better described by dependencies on amino acid volume, steric hindrance and FoldX predictions (see next subsection).

Another example is the solvent-exposed Gln205 (Fig. 2I) which exhibits a dependence on hydrophobicity contrary to that of Leu51. Gln205 is selected for its hydrophilicity, probably to aid in solubilizing the protein. Notably in this case, like in a few others, the ΔΔG^stat^ values distribute in a cluster of high probability (ΔΔG^stat^ ≈ 0 to 1.75) and another of low probability (ΔΔG^stat^ ≈ 2.25 to 4). Accordingly, hydrophobicity acts as a discrete classifier (being < 0.5 for the tolerated amino acids and > 0.5 for amino acids with low probability) rather than as a variable for continuous modelling.

Asn170, where hydrophilic residues are also favored, provides a more interesting example (Fig. 2C). This residue is on the protein surface but just 20.1% of its surface is exposed, thus it is not clear whether it contributes to solubility. However, Asn170 forms a hydrogen bond to Glu166, an active site residue, and could thus play an auxiliary catalytic role modulating the pKa and/or orientation of its carboxylate group (Fig. 3B). This is expected to be an important constraint, and indeed, the native amino acid is an outlier itself in the plot against hydrophobicity, being much more preferred than any other amino acid. This offset is observed in many correlations, suggesting that the wild type residues are largely preferred due to very specific reasons at some locations, although they can be substituted under certain constraints. In this example, Asn170 might be replaced by other residues that will not form exactly the same hydrogen bond with Glu166 but will at least preserve the polarity of the region. Notice that the composite variable *Hydrophobicity* x *Flexibility* explains slightly better the ΔΔG^stat^ distribution for Asn170, which could account for a secondary need for rigidity to better position the side chains for hydrogen bond formation.

The next two cases are examples of unsuspected relationships that this analysis helped unveil. First, Glu281 correlates positively with *Volume/P(helix)* (Fig. 2E) and negatively with *P(helix)/P(sheet)* (Fig. 2F). Other properties, such as hydrophobicity or solubility, do not correlate well (r^2^ = 0.1879 and 0.040, respectively) although they would be hypothesized to be important given that this residue’s side chain is exposed. Instead, the detected dependences reflect helical constraints on the backbone conformation, which must be preferably achieved with small amino acids. In the second case, for Gly87, the two descriptors *Hydrophobicity* and *P(sheet)* explain equally well the observed distributions (Fig. 2G and 2H). This residue is located in a tight turn on the protein surface and is 42% exposed, suggesting that the negative correlation with hydrophobicity reflects its role in conferring solubility. Notice that glycine is an important outlier in the plot against *Hydrophobicity*, probably due to the important conformational constraint revealed by its correlation against *P(sheet)*. The finding of two important dependencies might point at the requirement of a malleable (*i*.*e*. capable of adopting several defined conformations) and polar amino acid at this position rather than simply a “flexible” one, because flexibility does not correlate as well (r^2^ = 0.24). Notice that although glycine residues are usually attributed a role in conferring flexibility (which is indeed observed for most glycines located in loops, see next subsection), our analysis suggests that this particular glycine would fulfill other roles.

Arg222 and Ser98 provide examples where the atomic composition of the side chain describes the observed distributions better than any physicochemical property. The guanidinum group of Arg222 forms multiple hydrogen bonds and salt bridges with the carboxylate groups of Asp214 and Asp233 and with three backbone oxygen atoms, which effectively closes a loop located at the protein surface (Fig. 3C). Thus, arginine (3 nitrogen atoms on the side chain) is by far the preferred residue, followed by histidine (2 N atoms) and then the other amino acids (Fig. 2L). Similarly, Ser98 is roughly as likely as Thr or Asn, with the three of them having one oxygen atom in the side chain, whereas Asp and Glu (2 oxygens) are more favored than any of those three, and amino acids with no oxygens in their side chains are the least favored (Fig. 2M). Ser98 is located in a small loop closed through extensive hydrogen bonds to its alcohol group, where carboxylate groups could accommodate more interactions.

### Major constraints focus on stability of the protein core and of superficial hydrophobic clusters, followed by constraints on salt bridges and polar interactions at the surface, flexibility and solubility

**Fig 4:**
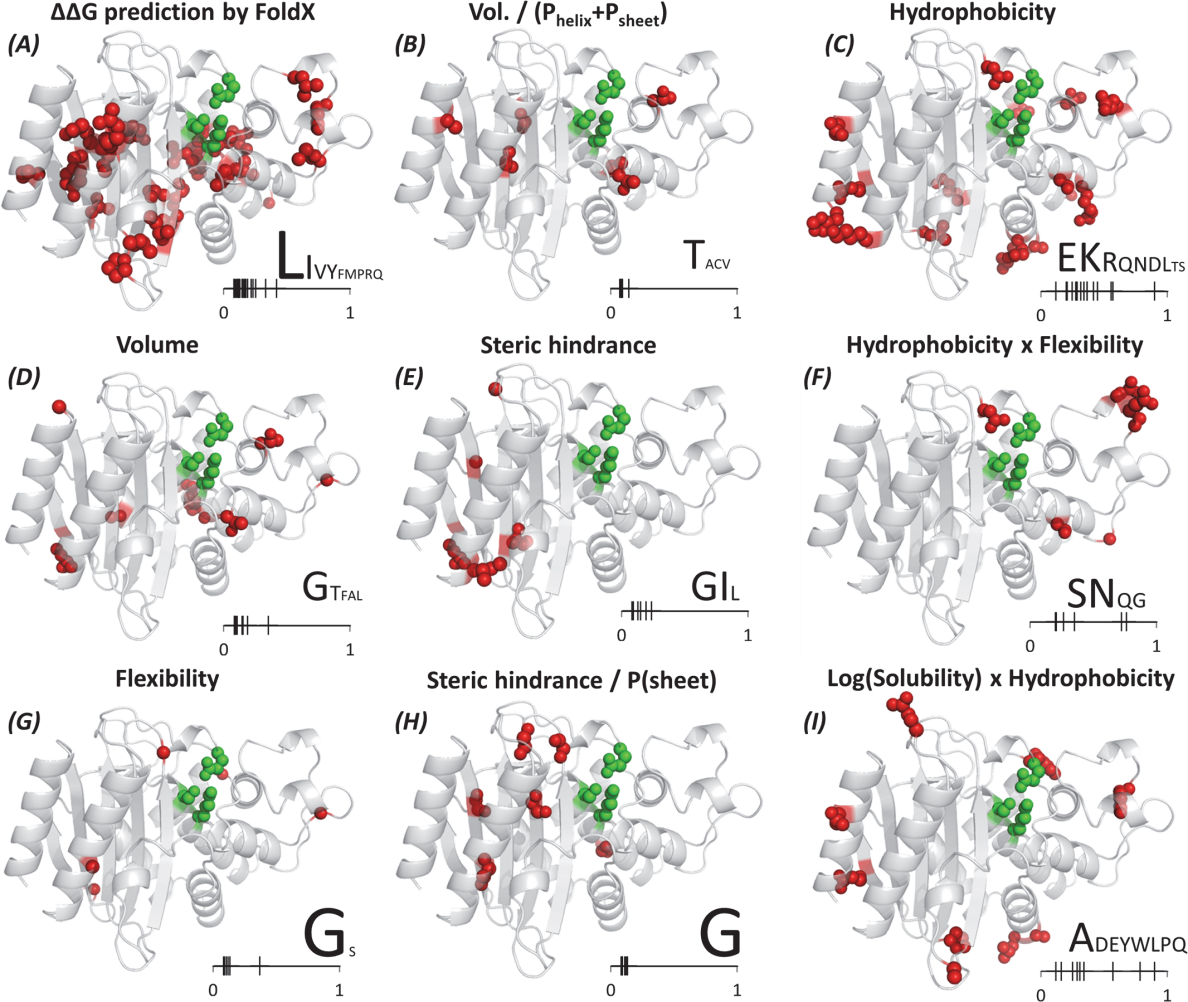
Structure mapping of the residues whose substitution patterns can be explained by the nine most common descriptors. The mapped amino acids are shown as red spheres, and residues Ser70, Lys73 and Glu166 as green spheres. All residue representations lack the main chain nitrogen, carbonyl carbon and oxygen atoms for clarity. The letters on the bottom right of each panel indicate the wild type amino acids most often found at the indicated locations, with the font size being roughly proportional to the relative number of occurrences of the amino acid. The small bar on the bottom right of each panel measures the fractional solvent exposure of the wild type residues to which the descriptor was mapped.

A more general interpretation of the results follows. For this, the descriptors most often selected are color-coded into the dots in Fig. 1 and mapped on TEM-1’s structure in Fig. 4. Different descriptors explain the ΔΔG^stat^ distributions (or in other words, shape the sequence constraints) at different locations of the protein. Correlations with ΔΔG^FoldX^ reflect the importance of hydrophobic clusters at the core (Fig. 4A and black dots in Fig. 1B) suggesting in turn that FoldX might perform particularly well for predictions of destabilization induced upon mutation deep inside protein cores. On top, correlations with *Volume/(P(helix)+P(sheet))*, *Volume*, *Steric hindrance* and *Steric hindrance/P(sheet)* mirror the need for small volumes and special residue packing at many locations inside the protein (Fig. 4B, D, E, H and Fig. 1B in cyan, blue, green, dark green, respectively). Finally, correlations against numbers of O or N atoms, *number of hydrogen bonds* or *Isoelectric point* map to residues involved in salt bridges and hydrogen bonds on the polar surface, like in the examples presented for Arg222 and Ser98.

Dependencies on *Hydrophobicity*, *Hydrophobicity x Flexibility* and *log(solubility) x Hydrophobicity* map to superficial sites of variable solvent exposure (Fig. 4C, F, I and Fig. 1B in red, magenta and orange, respectively). These residues would play roles in conferring solubility (cases where more polar or soluble amino acids are favored) and in compacting superficial hydrophobic clusters (more hydrophobic or less soluble amino acids). Six positions where flexibility was selected by the procedure (Fig. 4G) suggest an important contribution of dynamics at these locations, five of them actually mapping to loops. Moreover, three map to loops around the active site in the broad region that pivots the hinge motion linked to active site opening and closing as computed by normal mode analysis (S3 Fig.).

As a final note we would like to posit the idea that despite the analyzed data is specific for TEM-1 hydrolyzing ampicillin and thus most interpretations are valid under that setting, a significant fraction of the fits could reflect generalizable trends, especially those concerning protein stability and solubility.

### The unexplained distributions

We have thought of three main possible explanations for why no descriptors were retrieved for the ΔΔG^stat^ distributions of the remaining residues. First, as in the case of Thr195 described above, many positions have too low or too high k* and too little dispersion of the ΔΔG^stat^ values, which simply means that there is no trend to search for, or that a trend can be blurred by the uncertainties in ΔΔG^stat^. Indeed, low standard deviations in the ΔΔG^stat^ values are associated with either very low or very high k* values (S4 Fig.), implying large stringency or large tolerance to substitutions at those positions, respectively. We estimate that ∼ 10% of the protein sites are under such situation.

A second important difficulty in finding descriptors to account for ΔΔG^stat^ distributions might arise from multiple constraints that affect several properties simultaneously, and/or that follow non-monotonic dependencies on the descriptor(s) such that intermediate (rather than minimum or maximum) values are optimal. In principle, fitting to nonlinear functions and combinations of descriptors could unveil these patterns, but it would be difficult to assess the statistical significance of different fits. By focusing only on monotonic dependencies, we have concentrated on strong effects. Therefore, our analysis was limited only to the monotonic dependencies presented above, together with the good FoldX predictions. In a test protocol fitting unexplained ΔΔG^stat^ distributions to quadratic equations, which could roughly point at optimal values of a descriptor in which ΔΔG^stat^ is maximized or minimized, it was found that an additional 10–15% of distributions could be explained (examples of such fits are shown in S5 Fig.).

Finally, ΔΔG^stat^ distributions may be complicated by effects that do not mirror the inherent physics and chemistry of the protein, as discussed in literature [11,19] For example, codon substitutions could affect transcription or translation leading to premature transcription stops or ribosome stalling. Furthermore, effects on protein stability could be impacted by contributions from differential sensitivity to proteases. From what is left after the approximations given above, these undefined effects would apply to roughly 40–45% of the TEM-1 sequence positions.

## Concluding Remarks

The analysis presented here highlights the fundamental factors that shape an enzyme’s tolerance to substitutions, which ultimately dictate its accessible sequence space. These relationships are tightly linked to the principles of protein engineering and protein evolution. In particular, these analyses detect which protein trait(s) are expected to be sacrificed upon mutation either in an evolutionary setting or during rational design, beyond stability as usually recognized. For example, substitution of Gly238 by Ser, as observed in many extended-spectrum TEM variants, produces a decrease in flexibility, which is the most important property at that position (Fig. 2, panel O). If the detriment is too strong, the substitution might need to be buffered or compensated by additional substitutions. Regarding protein design, the analysis also suggests quantitative guidelines on where not to incorporate substitutions, which residues should generally be avoided when designing mutations, and which properties must be considered when designing substitutions based on the location of the residue in the protein structure. Our analysis does not allow us to unambiguously distinguish contributions to general traits (such as stability or solubility) from those to specific traits (such as effects on substrate binding or on the catalytic mechanism), although many observations could be rather general considering what is known about protein physical chemistry. In this regard, it would be very interesting and informative to analyze a similar dataset acquired under selection against a different β-lactam.

β-lactamases have been a particularly interesting target of structural biology studies, stemming from their clinical relevance and from a large experience accumulated on them, with tens to hundreds of characterized variants [41–46]. But as shown here and in the original work by Deng et al., competition experiments taking advantage of deep sequencing technologies can provide much larger amounts of information, and under homogeneous backgrounds and laboratory conditions. We expect that analyses of the kind introduced here will allow extraction of the fine structure information available in future datasets. Notably, no results like those reported here could be obtained from an alignment of natural TEM sequences, probably because of a lack of variability and/or confounding effects from epistatic interactions, or from other deep sequencing datasets of lower resolution [19,20]. The specific problem of epistatic interactions cannot be addressed with the available data, but deep sequencing technologies applied to large libraries of pairwise substitutions could shed light on how it builds up from non-additive effects of mutations. This is hard to test from low-throughput experiments or from natural variability but is an essential element of protein evolution and engineering. Our study demonstrates the power of deep sequencing experiments, particularly when large numbers of sequence reads are obtained.

## Methods

The dataset analyzed here corresponds exactly to that in ref. [18], which expands on a study at lower resolution as feasible two decades ago [47]. Specifically, the dataset was built by performing deep sequencing on a plasmid library with nearly full randomization of two to four contiguous codons, selected against 1 mg/ml ampicillin in *E*. *coli*. Such substitution scheme resulted in relatively smooth variations of the determined tolerance to substitutions at each single residue, thus enabling the detection of trends against amino acid descriptors (in contrast, very few correlations were retrieved from another study based on exclusively single substitutions).

For the analysis of ΔΔG^stat^ distributions in terms of amino acid properties, 28 descriptors were compiled in three sets from values available in references [36,37]. The first set contains five simple, uncorrelated properties (r^2^ < 0.3): *Volume*, *log(Solubility)*, *Hydrophobicity*, *Isoelectric point*, and *Helix propensity* (*P(helix)*). The second set contains another 17 descriptors, including simple ones that are correlated with those in the first set or with each other by 0.3 < r^2^ < 0.61, the products and ratios of descriptors from the first set (“composite variables”), and discrete descriptors reflecting the atomic composition of the side chain (number of O atoms, number of N atoms) and the maximum number of possible hydrogen bonds. The third set contains six additional simple and composite properties correlated by 0.61 < r^2^ < 0.72 to those in the first two sets. The inclusion of composite variables aims at accounting for ΔΔG^stat^ distributions of sites that are similarly sensitive to two different properties. All the 28 descriptors, their sources, values for each amino acid and correlations to each other are given in Supporting S1 Table.

The search for descriptors that could explain ΔΔG^stat^ distributions at each site was implemented as MS Excel and Matlab scripts. The procedure follows three incremental stages. First, we searched for correlations with the five basic properties (*i*.*e*. from the simplest set described above). Next, we searched for correlations with variables of the second set only for sites that did not correlate sufficiently well with any property from the first set. The same was then done with properties from the third set. The aim of this protocol was to minimize the inclusion of the more complex descriptors when correlations to simpler variables could explain the ΔΔG^stat^ distributions, yet allow them to be incorporated when no simpler descriptor was sufficient. In all cases, a descriptor was selected if it produced correlations better than 0.67 (r^2^ > 0.45) and RMSD < 1 between experimental and linearly back-predicted ΔΔG^stat^ values. The best and second-best descriptors selected for each position are listed in S2 Table. Notice that in some cases the correlation plots suggest dependences other than linear, but it is hard to assess with statistical significance the true underlying model.

Scanning the first set of five variables led to the substitution patterns for 26 positions correlating with a variable: 16 with hydrophobicity, eight with volume and two with isoelectric point (no positions correlated with *P(helix)* or *log(solubility)*). Extending the analysis to the second set of descriptors, correlations were found for a total of 98 positions; and including also the third set yielded a total of 105 correlations, carried on to the analysis presented under Results and Discussion. All the correlations can be explored interactively in the spreadsheet provided in the supporting material (S2 Table).

## Supporting Information

S1 Fig Distribution of picked descriptors. Distribution of picked descriptors. across the 105 correlations with amino acid properties (FoldX predictions not included).
(DOCX)

S2 Fig Sequence distribution of descriptors. Sequence distribution of descriptors picked to explain the observed ΔΔG^stat^ values at each site and of sites where ΔΔG^stat^ correlates with ΔΔG^FoldX^, together with plots showing the distance to the active site and the fraction of exposed area for the wild type residue (both extracted from PDB ID 1XPB). When two descriptors were found, the second-best is shown on top.
(DOCX)

S3 Fig Open and closed conformations of TEM-1. Conformations as retrieved from normal mode analysis on the 1XPB structure (second mode computed with ProDy with a 15 Å cutoff). Spheres map to the Cα atoms of all glycines; green spheres map to residues for which flexibility was selected as the main descriptor explaining ΔΔG^stat^. Picture rendered in VMD.
(DOCX)

S4 Fig Plot of the standard deviation in ΔΔG^stat^ versus k*. Plot of the standard deviation in ΔΔG^stat^ versus k* for all the residues in mature TEM. Green dots correspond to residues whose distribution was explained by the descriptors; red dots are the unexplained residues.
(DOCX)

S5 Fig Examples of fits to quadratic dependencies. Four examples of fits to quadratic dependencies on the descriptors, which satisfy r^2^ > 0.45 and RMSD < 1 between experimental and back-predicted ΔΔG^stat^. Notice that these plots do not necessarily imply a strict quadratic dependence on the variable. For example, the plot for Lys73 seems to point out that only very polar residues are allowed, while the plot for Gly143 could point out that only amino acids with very low beta sheet propensity are allowed. On the other hand, the plots for Ser82 and Thr266 seem to truly reflect an optimal balance between two main amino acid properties.
(DOCX)

S1 Table Amino acid descriptors employed in this work. The 28 amino acid descriptors compiled here, which were scanned to find correlations with ΔΔG^stat^ distributions. The five simplest properties are shown in dark green background, the second set in orange, the third one in pink. Correlations between all descriptors are given.
(XLSX)

S2 Table Interactively scanning descriptors for correlations with ΔΔG^stat^ data. Interactive spreadsheet in which the 28 descriptors can be scanned for the best and second-best correlations. Clicking “up” and “down” moves through the protein sequence. Only cases for which r^2^ > 0.45 and RMSD < 1 are displayed. This file is compatible with Microsoft Excel version 2010 and up (active content must be enabled), but not with Excel for Mac OS.
(XLSM)

S3 Table List of descriptors selected by the scanning procedure. List of TEM-1 residues showing the best descriptor accounting for each ΔΔG^stat^ distribution (labeled “1”), the second-best descriptor (“2”) and good correlations with ΔΔG^FoldX^.
(XLSX)

S4 Table Interactive testing of ΔΔG^stat^ fits against ΔΔG^FoldX^ predictions. Interactive spreadsheet with which the ΔΔG^stat^ values can be compared against ΔΔG^FoldX^ predictions. Clicking “up” and “down” moves through the protein sequence. This file is compatible with Microsoft Excel version 2010 and up (active content must be enabled), but not with Excel for Mac OS.
(XLSM)
