# Supplementary material for: How Structural and Physicochemical Determinants Shape Sequence Constraints in a Functional Enzyme
Source: PLoS One. 2015 Feb 23;10(2):e0118684. doi: 10.1371/journal.pone.0118684 (PMC4338278; doi:10.1371/journal.pone.0118684)
Supplement: S1 Fig — Distribution of picked descriptors. across the 105 correlations with amino acid properties (FoldX predictions not included). (DOCX) [file pone.0118684.s001.docx]

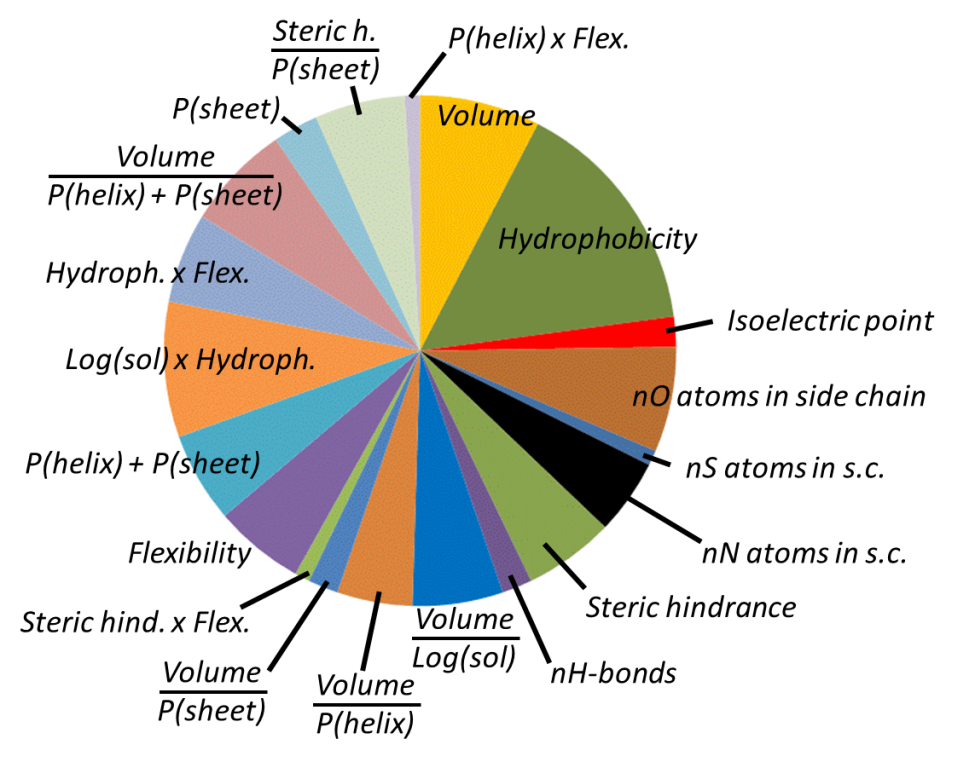


**Fig. S1.** Distribution of picked descriptors across the 105 correlations with amino acid properties (FoldX predictions not included).
