# Supplementary material for: How Structural and Physicochemical Determinants Shape Sequence Constraints in a Functional Enzyme
Source: PLoS One. 2015 Feb 23;10(2):e0118684. doi: 10.1371/journal.pone.0118684 (PMC4338278; doi:10.1371/journal.pone.0118684)
Supplement: S2 Fig — Sequence distribution of descriptors picked to explain the observed ΔΔGstat values at each site and of sites where ΔΔGstat correlates with ΔΔGFoldX, together with plots showing the distance to the active site and the fraction of exposed area for the wild type residue (both extracted from PDB ID 1XPB). When two descriptors were found, the second-best is shown on top. (DOCX) [file pone.0118684.s002.docx]

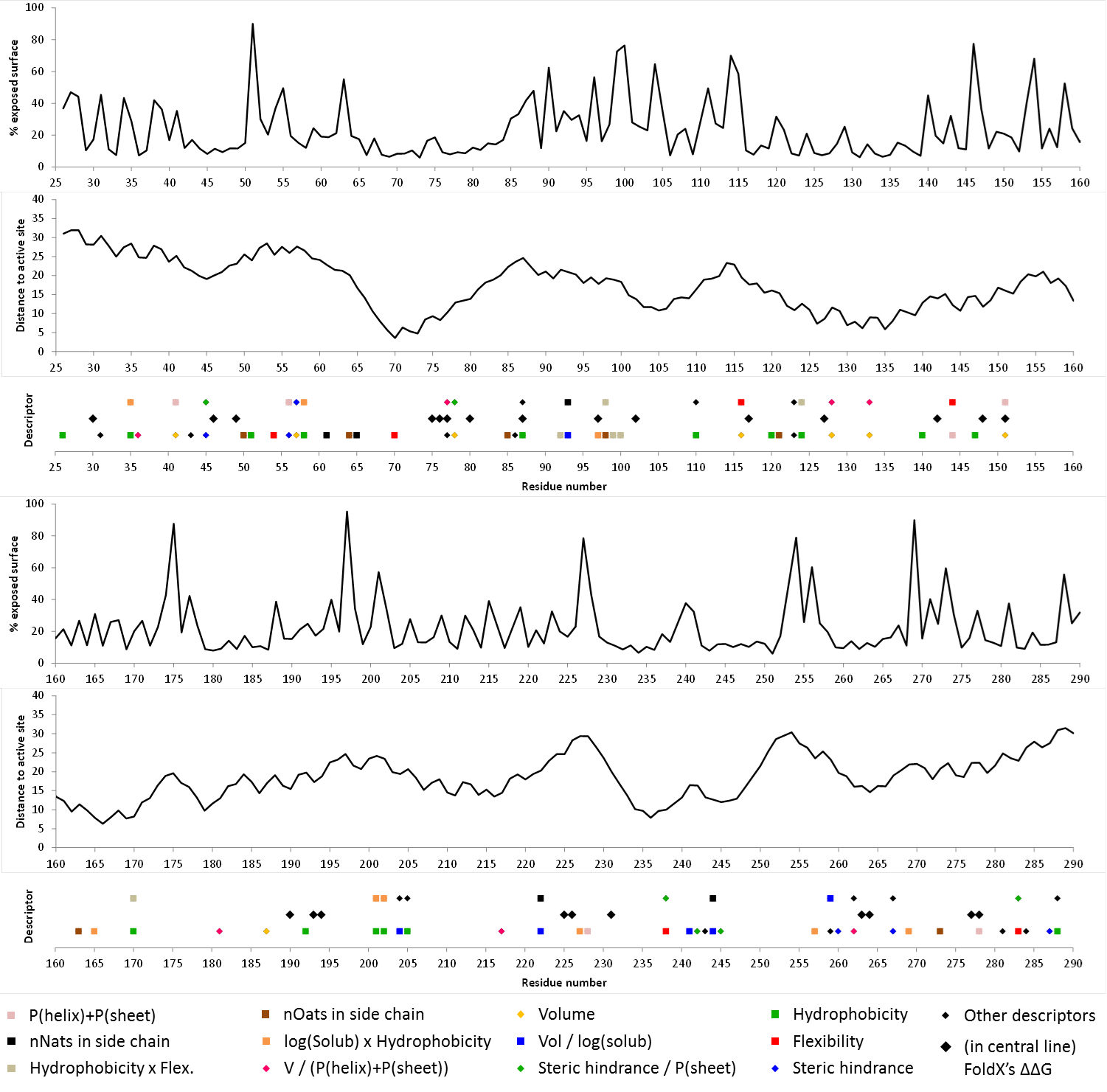


**Fig. S2.** Sequence distribution of descriptors picked to explain the observed ΔΔG^stat^ values at each site and of sites where ΔΔG^stat^ correlates with ΔΔG^foldx^, together with plots showing the distance to the active site and the fraction of exposed area for the wild type residue (both extracted from PDB ID 1XPB). When two descriptors were found, the second-best is shown on top.
