# Supplementary material for: How Structural and Physicochemical Determinants Shape Sequence Constraints in a Functional Enzyme
Source: PLoS One. 2015 Feb 23;10(2):e0118684. doi: 10.1371/journal.pone.0118684 (PMC4338278; doi:10.1371/journal.pone.0118684)
Supplement: S3 Fig — Conformations as retrieved from normal mode analysis on the 1XPB structure (second mode computed with ProDy with a 15 Å cutoff). Spheres map to the Cα atoms of all glycines; green spheres map to residues for which flexibility was selected as the main descriptor explaining ΔΔGstat. Picture rendered in VMD. (DOCX) [file pone.0118684.s003.docx]

**
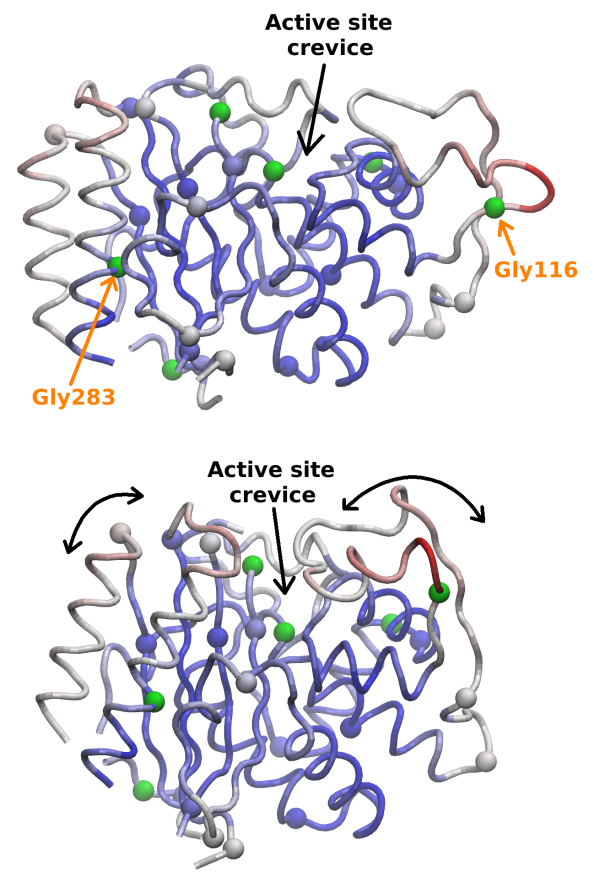
**

**Fig. S3.** Open and closed conformations of TEM-1 as retrieved from normal mode analysis on the 1XPB structure (second mode computed with ProDy with a 15 Å cutoff). Spheres map to the Cα atoms of all glycines; green spheres map to residues for which flexibility was selected as the main descriptor explaining ΔΔG^stat^. Picture rendered in VMD.
