# Supplementary material for: How Structural and Physicochemical Determinants Shape Sequence Constraints in a Functional Enzyme
Source: PLoS One. 2015 Feb 23;10(2):e0118684. doi: 10.1371/journal.pone.0118684 (PMC4338278; doi:10.1371/journal.pone.0118684)
Supplement: S4 Fig — Plot of the standard deviation in ΔΔGstat versus k* for all the residues in mature TEM. Green dots correspond to residues whose distribution was explained by the descriptors; red dots are the unexplained residues. (DOCX) [file pone.0118684.s004.docx]

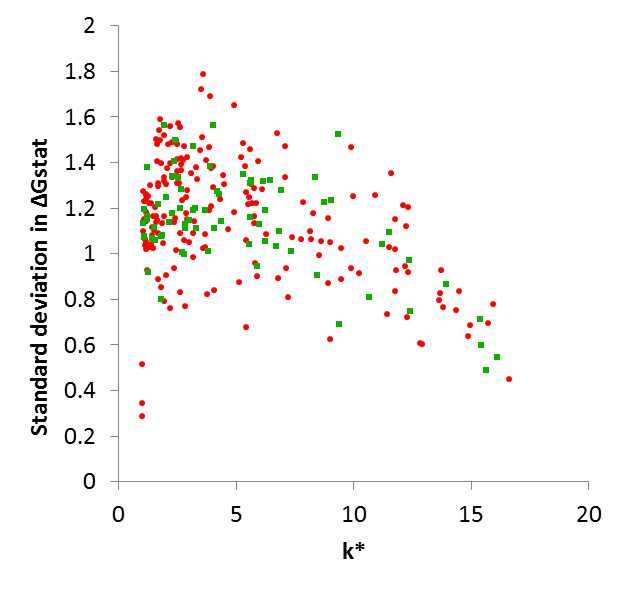


**Fig. S4.** Plot of the standard deviation in ΔΔG^stat^ versus *k** for all the residues in mature TEM. Green dots correspond to residues whose distribution was explained by the descriptors; red dots are the unexplained residues.
