# Supplementary material for: How Structural and Physicochemical Determinants Shape Sequence Constraints in a Functional Enzyme
Source: PLoS One. 2015 Feb 23;10(2):e0118684. doi: 10.1371/journal.pone.0118684 (PMC4338278; doi:10.1371/journal.pone.0118684)
Supplement: S5 Fig — Four examples of fits to quadratic dependencies on the descriptors, which satisfy r2 > 0.45 and RMSD < 1 between experimental and back-predicted ΔΔGstat. Notice that these plots do not necessarily imply a strict quadratic dependence on the variable. For example, the plot for Lys73 seems to point out that only very polar residues are allowed, while the plot for Gly143 could point out that only amino acids with very low beta sheet propensity are allowed. On the other hand, the plots for Ser82 and Thr266 seem to truly reflect an optimal balance between two main amino acid properties. (DOCX) [file pone.0118684.s005.docx]

**
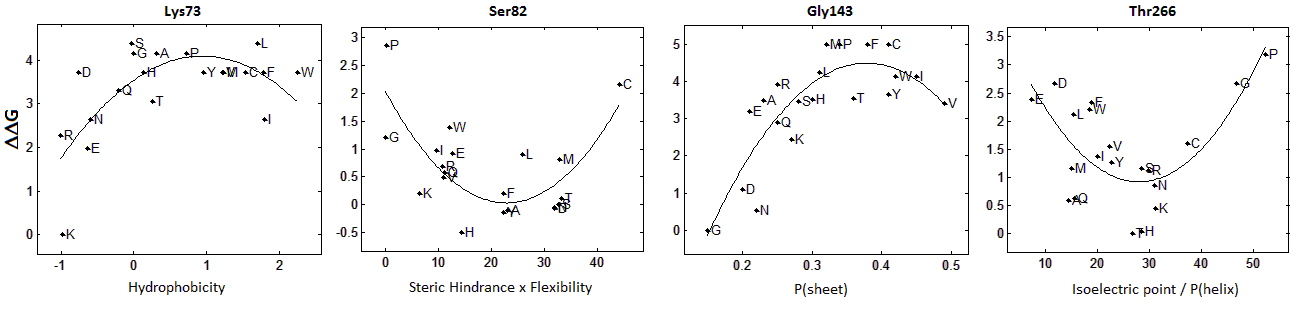
**

**Fig. S5.** Four examples of fits to quadratic dependencies on the descriptors, which satisfy r^2^ > 0.45 and RMSD < 1 between experimental and back-predicted ΔΔG^stat^. Notice that these plots do not necessarily imply a strict quadratic dependence on the variable. For example, the plot for Lys73 seems to point out that only very polar residues are allowed, while the plot for Gly143 could point out that only amino acids with very low beta sheet propensity are allowed. On the other hand, the plots for Ser82 and Thr266 seem to truly reflect an optimal balance between two main amino acid properties.
